# On the mixed Kibria–Lukman estimator for the linear regression model

**DOI:** 10.1038/s41598-022-16689-z

**Published:** 2022-07-20

**Authors:** Hongmei Chen, Jibo Wu

**Affiliations:** 1 College of Mathematics and Statistics, Chongqing Jiaotong University, Chongqing, China; 2 School of Mathematics and Big Data, Chongqing University of Arts and Sciences, Chongqing, China

**Keywords:** Applied mathematics, Statistics

## Abstract

This paper considers a linear regression model with stochastic restrictions,we propose a new mixed Kibria–Lukman estimator by combining the mixed estimator and the Kibria–Lukman estimator.This new estimator is a general estimation, including OLS estimator, mixed estimator and Kibria–Lukman estimator as special cases. In addition, we discuss the advantages of the new estimator based on MSEM criterion, and illustrate the theoretical results through examples and simulation analysis.

## Introduction

Consider the following linear regression model:

1 $$\begin{aligned} y=X\beta +\varepsilon , \end{aligned}$$

where *y* is the response variable vector of $n \times 1, X$ is the column full rank independent variables matrix of $n \times (p+1), \beta$ is the unknown coefficient vector of $p \times 1, \varepsilon$ is the random error vector of *n* dimension such that $E(\varepsilon )=0$ and ${\text {Cov}}(\varepsilon )=\sigma ^{2} I$, where $\sigma ^{2}>0$ is mean squared error.

In the estimation of unknown coefficient vector $\beta$, the OLS estimator is the most commonly used:

2 $$\begin{aligned} \hat{\beta }_{OLS}=\left( X^{\prime } X\right) ^{-1} X^{'}y \end{aligned}$$

It is easy to know from formula (2), $E \hat{\beta }=\beta$, and the OLS estimator has been widely used because of its unbiased nature and concise form. However, the ill condition of the design matrix *X* caused by the increasing number of dependent predictors often makes the OLS estimates unstable.

Massy^1^ proposed principal component estimator. Hoerl and Kennard^2^ obtained the ridge estimation by introducing a ridge parameter k into the design $X'X$ matrix calculation. Swindel^3^ proposed a modified ridge estimator with prior information while Lukman et al.^4^ proposed the two-parameter form of the ridge estimator called the modified ridge estimator (MRT). Liu^5^ obtained a linearized form of the ridge estimator called the Liu estimator. Akdeniz and Kaciranlara^6^ proposed the generalized Liu estimator. Liu^7^ obtained a two-parameter form of the Liu estimator.

Many scholars have found that a new estimator can be obtained by combining the two estimators, which generally have good statistical properties. Baye and Parker^8^ proposed r–k estimator by combining ridge estimator and principal component estimator. Kaciranlar and Sakallioglu^9^ proposed r–d estimator by combining Liu estimator and principal component estimator. Ozkale and Kaciranlar^10^ proposed two parameter estimator by combining the James–Stein Shrinkage estimator and the modified ridge estimator proposed by Swindel. Batah et al.^11^ proposed a modified r-k estimator combining unbiased ridge estimator and principal component estimator. Yang and Chang^12^ proposed another two parameter estimator based on ridge estimator and Liu estimator. Lukman et al.^13^ proposed a new estimator by combining modified ridge estimator (MRT) and principal component estimator. Kibria and Lukman^14^ proposed Kibria–Lukman estimator by combining ridge estimator and Liu estimator.

In practice, in addition to the sample information given by model (1), additional information about parameters in the sample information, such as certain deterministic or stochastic restrictions on unknown parameters, can also be considered. This method can also overcome the complex collinearity problem. Theil and Goldberger^15^ and Theil^16^ proposed mixed estimator by comprehensively considering sample information and constraints. Schiffrin and Toutenburg^17^ proposed weighted mixed estimator for the different importance of sample information and prior information.

In recent years, biased estimation and estimation methods with prior information are often combined to form a broader biased estimation. Hubert and Wijekoon^18^ proposed a stochastic restricted Liu estimator by combining Liu estimator and mixed estimator. Yang and Xu^19^ obtained another stochastic mixed Liu estimator. In the same year, Yang and Chang further studied the stochastic mixed Liu estimator and obtained the weighted mixed Liu estimator. Yang and Li^12^ proposed another stochastic mixed ridge estimator. Ozbay and Kaciranlar^20^ integrated two parameter estimator and mixed estimator and proposed a two parameter mixed estimator.

In this paper, a new mixed KL estimator under stochastic restrictions is proposed, and its excellent properties under certain conditions are proved theoretically. The above theoretical results are verified and analyzed by examples and data simulation.

## The proposed estimator

Hoerl and Kennard^2^ proposed the ridge estimator (RE):

3 $$\begin{aligned} \hat{\beta }_{R E}=\left( X^{\prime } X+k I\right) ^{-1} X^{\prime } y \end{aligned}$$

where $k>0$ is the parameter. In fact, ridge estimator is obtained by solving the following extreme value problem:

$$\begin{aligned} (y-X \beta )^{\prime }(y-X \beta )+k\left( \beta ^{\prime } \beta -c\right) \end{aligned}$$

where *c* is constant, *k* is the Lagrange constant.

Kibria and Lukman^14^ proposed the Kibria Lukman (KL) estimator:

4 $$\begin{aligned} \hat{\beta }_{K L}=\left( X^{\prime } X+k I\right) ^{-1}\left( X^{\prime } y-k \hat{\beta }\right) \end{aligned}$$

where $k>0$ is the parameter.KL estimator is obtained by solving the following extreme value problem:

5 $$\begin{aligned} (y-X \beta )^{\prime }(y-X \beta )+k\left[ (\beta +\hat{\beta })^{\prime }(\beta +\hat{\beta })-c\right] \end{aligned}$$

where *c* is constant, *k* is the Lagrange constant.

Consider the following stochastic restrictions:

6 $$\begin{aligned} r=R \beta +e, e \sim \left( 0, \sigma ^{2} \psi \right) , \end{aligned}$$

where *r* is the known random vector of $j \times 1$, *R* is the row full rank sample data matrix of $j \times p$, let *e* be the $j \times 1$ random error vector and independent of each other, and $\psi$ be the known positive definite matrix.

Theil and Goldberger^15^ and Theil^16^ proposed the mixed estimator by integrating sample information and constraints. The derivation idea is to rewrite models (1) and (6) into a new linear model:

$$\begin{aligned} \left( \begin{array}{l} y \\ r \end{array}\right) =\left( \begin{array}{l} X \\ R \end{array}\right) \beta +\left( \begin{array}{l} \varepsilon \\ e \end{array}\right) \end{aligned}$$

If $$\tilde{y}=\left( \begin{array}{l}y \\ r\end{array}\right) , \tilde{X}=\left( \begin{array}{l}X \\ R\end{array}\right) , \tilde{\varepsilon }=\left( \begin{array}{l}\varepsilon \\ e\end{array}\right)$$, above model is transformed into

7 $$\begin{aligned} \tilde{y}=\tilde{X} \beta +\tilde{\varepsilon } \end{aligned}$$

By applying the least square estimator to the new linear model (7), the mixed estimator (ME)of parameter $\beta$ is obtained:

8 $$\begin{aligned} \hat{\beta }_{M E}=\left( X^{\prime } X+R^{\prime } \psi ^{-1} R\right) ^{-1}\left( X^{\prime } y+R^{\prime } \psi ^{-1} r\right) \end{aligned}$$

Combined mixed estimator and ridge estimator and proposed stochastic mixed ridge estimation (RME):

9 $$\begin{aligned} \hat{\beta }_{MRE}=\left( X^{\prime } X+k I+R^{\prime } \psi ^{-1} R\right) ^{-1}\left( X^{\prime } y+R^{\prime } \psi ^{-1} r\right) \end{aligned}$$

The estimator proposed in this paper is obtained by solving the following extreme value problem:

10 $$\begin{aligned} \Phi ^{*}=(y-X \beta )^{\prime }(y-X \beta )+k\left[ (\beta -d \hat{\beta })^{\prime }(\beta -d \hat{\beta })-c\right] +(r-R \beta )^{\prime } \psi ^{-1}(r-R \beta ) \end{aligned}$$

where *c* is constant, *k* is Lagrange constant.

Regular equations can be obtained:

11 $$\begin{aligned}&X^{'}X \beta -X^{'}y+k(\beta -d \hat{\beta })+R^{\prime } \psi ^{-1} R-R^{\prime } \psi ^{-1} r=0 \end{aligned}$$

12 $$\begin{aligned} (\beta -d \hat{\beta })^{\prime }(\beta -d \hat{\beta })=c \end{aligned}$$

from Eqs. (11) and (12), we can get the mixed KL estimator:

13 $$\begin{aligned} \hat{\beta }_{MKL}=\left( X^{'}X+k I+R^{\prime } \psi ^{-1} R\right) ^{-1}\left( X^{'}y-k \hat{\beta }+R^{\prime } \psi ^{-1} r\right) , k>0 \end{aligned}$$

It can be seen from Eq. (13) that mixed estimator, KL estimator and OLS estimator can be regarded as special cases of mixed KL estimator.Namely

When $k=0, \hat{\beta }_{ME}=\hat{\beta }_{MKL}=\left( X^{\prime } X+R^{\prime } \psi ^{-1} R\right) ^{-1}\left( X^{\prime } y+R^{\prime } \psi ^{-1} r\right)$ is mixed estimator;

When $R=0, \hat{\beta }_{K L}=\hat{\beta }_{MKL}=(X^{'}X+k I)^{-1}(X'y-k \hat{\beta })$ is $\mathrm {KL}$ estimator;

When $k=0, R=0, \hat{\beta }_{OLS}=\hat{\beta }_{MKL}=(X^{'}X)^{-1}X^{'}y$ is OLS estimator.

## The performance of the new estimator

If $\hat{\beta }$ is the estimation of $\beta$, then the mean square error matrix $(\mathrm {MSEM})$ of $\hat{\beta }$ is given as:

$$\begin{aligned} {\text {MSEM}}(\hat{\beta })=E(\hat{\beta }-\beta )(\hat{\beta }-\beta )^{\prime }={\text {Cov}}(\hat{\beta })+{\text {Bias}}(\hat{\beta }) {\text {Bias}}(\hat{\beta })^{'} \end{aligned}$$

where ${\text {Cov}}(\hat{\beta })$ is the covariance matrix of $\hat{\beta }$, and ${\text {Bias}}(\hat{\beta })=E(\hat{\beta })-\beta$ is the deviation vector. Two estimates $\hat{\beta }_{1}$ and $\hat{\beta }_{2}$, $\hat{\beta }_{2}$ are better than $\hat{\beta }_{1}$ under MSEM criterion if and only if:

$$\begin{aligned} \Delta \left( \hat{\beta }_{1}, \hat{\beta }_{2}\right) ={\text {MSEM}}\left( \hat{\beta }_{1}\right) -{\text {MSEM}}\left( \hat{\beta }_{2}\right) \ge 0 \end{aligned}$$

### Lemma 3.1

*Suppose two* $n \times n$ *matrix* $M>0, N \ge 0$, *then* $M>N \Leftrightarrow \lambda _{1}\left( N M^{-1}\right) <1$, *where* $\lambda _{1}\left( N M^{-1}\right)$ *is the maximum eigenvalue of matrix* $N M^{-1}$.

*The mean square error matrix of mixed KL estimator* $\hat{\beta }_{MKL}$ *is calculated as follows:*

14 $$\begin{aligned} E\left( \hat{\beta }_{MKL}\right)= \, & {} E\left[ \left( X^{\prime } X+k I+R^{\prime } \psi ^{-1} R\right) ^{-1}\left( X^{'}y-k \hat{\beta }+R^{\prime } \psi ^{-1} r\right) \right] \nonumber \\= \, & {} A_{k} E\left( X^{'}y-k \hat{\beta }+R^{\prime } \psi ^{-1} r\right) \nonumber \\= \, & {} A_{k} E\left( X^{'}y+k \hat{\beta }-2 k \hat{\beta }+R^{\prime } \psi ^{-1} r\right) \nonumber \\= \, & {} A_{k}\left( A_{k}^{-1}-2 k\right) \beta \nonumber \\= \, & {} \beta -2 k A_{k} \beta \end{aligned}$$

*where* $$A_{k}=\left( X^{'}X+k I+R^{\prime } \psi ^{-1} R\right) ^{-1}$$.

*Deviation vector:* $${\text {Bias}}\left( \hat{\beta }_{MKL}\right) =E\left( \hat{\beta }_{MKL}\right) =-2 k A_{k} \beta$$.

15 $$\begin{aligned} {\text {Cov}}\left( \hat{\beta }_{MKL}\right)= \, & {} {\text {Cov}}\left[ \left( X^{\prime } X+k I+R^{\prime } \psi ^{-1} R\right) ^{-1}\left( X^{'}y-k \hat{\beta }+R^{\prime } \psi ^{-1} r\right) \right] \nonumber \\= \, & {} {\text {Cov}}\left[ A_{k}\left( X^{'}y-k \hat{\beta }+R^{\prime } \psi ^{-1} r\right) \right] \nonumber \\= \, & {} A_{k} {\text {Cov}}\left( X^{'}y-k \hat{\beta }+R^{\prime } \psi ^{-1} r\right) A_{k}\nonumber \\= \, & {} A_{k}\left( \sigma ^{2} X^{\prime } X-k \sigma ^{2} S^{-1}+\sigma ^{2} R^{\prime } \psi ^{-1} R\right) A_{k}\nonumber \\= \, & {} \sigma ^{2} A_{k}\left( X^{\prime } X-k S^{-1}+R^{\prime } \psi ^{-1} R\right) A_{k} \end{aligned}$$

*Therefore,*

16 $$\begin{aligned} {\text {MSEM}}(\hat{\beta }_{MKL})= \, & {} {\text {Cov}}(\hat{\beta }_{MKL})+{\text {Bias}}(\hat{\beta }_{MKL}) {\text {Bias}}(\hat{\beta }_{MKL})^{'}\nonumber \\= \, & {} \sigma ^{2} A_{k}\left( X^{\prime } X-k S^{-1}+R^{\prime } \psi ^{-1} R\right) A_{k}+4 k^{2} A_{k} \beta \beta ^{\prime } A_{k}\nonumber \\= \, & {} \sigma ^{2} A_{k}\left( X^{\prime } X-k S^{-1}+R^{\prime } \psi ^{-1} R\right) A_{k}+b_{1} b_{1}^{\prime } \end{aligned}$$

*where* $$b_{1}=-2 k A_{k} \beta .$$

*By substituting* $k=0$ *into Eq.* (16), *the mean square error matrix of the mixed estimator can be obtained:*

17 $$\begin{aligned} {\text {MSEM}}(\hat{\beta }_{ME})= \, & {} \sigma ^{2}\left( X^{\prime } X+R^{\prime } \psi ^{-1} R\right) ^{-1}\left( X^{\prime } X+R^{\prime } \psi ^{-1} R\right) \left( X^{\prime } X+R^{\prime } \psi ^{-1} R\right) ^{-1}\nonumber \\= \, & {} \sigma ^{2}\left( X^{\prime } X+R^{\prime } \psi ^{-1} R\right) ^{-1}\nonumber \\= \, & {} \sigma ^{2} M^{-1} \end{aligned}$$

*where* $$M=X^{\prime } X+R^{\prime } \psi ^{-1} R$$.

*By substituting* $R=0$ *into Eq.* (16), *the mean square error matrix of the KL estimator can be obtained:*

18 $$\begin{aligned} {\text {MSEM}}\left( \hat{\beta }_{KL}\right)= \, & {} \sigma ^{2}\left( X^{\prime } X+k I\right) ^{-1}\left( X^{\prime } X-k S^{-1}\right) \left( X^{\prime } X+k I\right) ^{-1}\nonumber \\\, &\,+4 k^{2}\left( X^{\prime } X+k I\right) ^{-1} \beta \beta ^{\prime }\left( X^{\prime } X+k I\right) ^{-1}\nonumber \\= \, & {} \sigma ^{2} S_{k}^{-1}\left( X^{\prime } X-k S^{-1}\right) S_{k}^{-1}+4 k^{2} S_{k}^{-1} \beta \beta ^{\prime } S_{k}^{-1}\nonumber \\= \, & {} \sigma ^{2} S_{k}^{-1}\left( X^{\prime } X-k S^{-1}\right) S_{k}^{-1}+b_{2} b_{2}^{\prime } \end{aligned}$$

*where* $$S_{k}=X^{\prime } X+k I, b_{2}=-2 k S_{k}^{-1} \beta$$.

*By substituting* $k=0, R=0$ *into Eq.* (16), *the mean square error matrix of the OLS estimator can be obtained:*

19 $$\begin{aligned} {\text {MSEM}}\left( \hat{\beta }_{\text{ OLS } }\right) =\sigma ^{2} S^{-1} \end{aligned}$$

*Mean square error matrix of mixed ridge estimator:*

20 $$\begin{aligned} E\left( \hat{\beta }_{MRE}\right)= \, & {} E\left[ \left( X^{\prime } X+k I+R^{\prime } \psi ^{-1} R\right) ^{-1}\left( X^{'}y+R^{\prime } \psi ^{-1} r\right) \right] \nonumber \\= \, & {} A_{k} E\left( X^{\prime } y+R^{\prime } \psi ^{-1} r\right) \nonumber \\= \, & {} A_{k} E\left( X^{'}y+k \hat{\beta }-k \hat{\beta }+R^{\prime } \psi ^{-1} r\right) \nonumber \\= \, & {} A_{k}\left( A_{k}^{-1}-kI\right) \beta \nonumber \\= \, & {} \beta -k A_{k} \beta \end{aligned}$$

*Deviation vector:* $${\text {Bias}}\left( \hat{\beta }_{MRE}\right) =E\left( \hat{\beta }_{MRE}\right) -\beta =-k A_{k} \beta .$$

$$\begin{aligned} \begin{aligned} Cov\left( \hat{\beta }_{MRE}\right)&=\, {\text {Cov}}\left[ \left( X^{\prime } X+k I+R^{\prime } \psi ^{-1} R\right) ^{-1}\left( X^{\prime } y+R^{\prime } \psi ^{-1} r\right) \right] \\&=\,{\text {Cov}}\left[ A_{k}\left( X^{'}y+R^{\prime } \psi ^{-1} r\right) \right] \\&=A_{k} {\text {Cov}}\left( X^{'}y+R^{\prime } \psi ^{-1} r\right) A_{k}\\&=A_{k}\left( \sigma ^{2} X^{\prime } X+\sigma ^{2} R^{\prime } \psi ^{-1} R\right) A_{k} \\&=\,\sigma ^{2} A_{k}\left( X^{\prime } X+R^{\prime } \psi ^{-1} R\right) A_{k} \end{aligned} \end{aligned}$$

*Therefore,*

21 $$\begin{aligned} {\text {MSEM}}\left( \hat{\beta }_{MRE}\right) =\sigma ^{2} A_{k}\left( X^{\prime } X+R^{\prime } \psi ^{-1} R\right) A_{k}+k^{2} A_{k} \beta \beta ^{\prime } A_{k}. \end{aligned}$$

### Comparison between mixed KL estimator and mixed estimator

From Eqs. (16) and (17), we make

22 $$\begin{aligned} \Delta _{1}= \, & {} {\text {MSEM}}\left( \hat{\beta }_{M E}\right) -{\text {MSEM}}\left( \hat{\beta }_{M K L}\right) \nonumber \\= \, & {} \sigma ^{2} M^{-1}-\sigma ^{2} A_{k}\left( X^{\prime } X-k S^{-1}+R^{\prime } \psi ^{-1} R\right) A_{k}-b_{1} b_{1}^{\prime }\nonumber \\= \, & {} \sigma ^{2} M^{-1}-\sigma ^{2} A_{k}\left( M-k S^{-1}\right) A_{k}-b_{1} b_{1}^{\prime }\nonumber \\= \, & {} \sigma ^{2}\left[ M^{-1}-A_{k}\left( M-k S^{-1}\right) A_{k}\right] -b_{1} b_{1}^{\prime } \end{aligned}$$

*Because*

$$\begin{aligned}&M^{-1}-A_{k}\left( M-k S^{-1}\right) A_{k}\\&\quad =A_{k} A_{k}^{-1} M^{-1} A_{k}^{-1} A_{k}-A_{k}\left( M-kS^{-1}\right) A_{k}\\&\quad =A_{k}\left[ A_{k}^{-1} M^{-1} A_{k}^{-1}-\left( M-k S^{-1}\right) \right] A_{k} \\&\quad =A_{k}\left[ (M+k I) M^{-1}(M+k I)-\left( M-k S^{-1}\right) \right] A_{k} \\&\quad =A_{k}\left( M+2 k I+k^{2} M^{-1}-M+k S^{-1}\right) A_{k} \\&\quad =A_{k}\left( 2 k I+k^{2} M^{-1}+k S^{-1}\right) A_{k}, \end{aligned}$$

from $k>0$,so $M^{-1}-A_{k}\left( M-k S^{-1}\right) A_{k}>0$, Theorem 3.2 is obtained.

#### Theorem 3.2

*The necessary and sufficient conditions for mixed KL estimator* $\hat{\beta }_{MKL}$ *to be superior to mixed estimator* $\hat{\beta }_{M E}$ *under MSEM criterion are as follows*:

23 $$\begin{aligned} \sigma ^{-2} b_{1}^{\prime }\left[ M^{-1}-A_{k}\left( M-k S^{-1}\right) A_{k}\right] ^{-1} b_{1} \le 1 \end{aligned}$$

### Comparison between mixed KL estimator and KL estimator

From Eqs. (16) and (18), we make

24 $$\begin{aligned} \begin{aligned} \Delta _{2}&={\text {MSEM}}\left( \hat{\beta }_{KL}\right) -{\text {MSEM}}\left( \hat{\beta }_{MKL}\right) \\&=\sigma ^{2} S_{k}^{-1}\left( S-k S^{-1}\right) S_{k}^{-1}+b_{2} b_{2}^{\prime }-\sigma ^{2} A_{k}\left( S-k S^{-1}+R^{\prime } \psi ^{-1} R\right) A_{k}-b_{1} b_{1}^{\prime } \\&=\sigma ^{2}\left[ S_{k}^{-1}\left( S-k S^{-1}\right) S_{k}^{-1}-A_{k}\left( S-k S^{-1}+R^{\prime } \psi ^{-1} R\right) A_{k}\right] +b_{2} b_{2}^{\prime }-b_{1} b_{1}^{\prime } \end{aligned} \end{aligned}$$

*Because*

$$\begin{aligned}&S_{k}^{-1}\left( S-k S^{-1}\right) S_{k}^{-1}-A_{k}\left( S-k S^{-1}+R^{\prime } \psi ^{-1} R\right) A_{k} \\&\quad =A_{k}\left[ A_{k}^{-1} S_{k}^{-1}\left( S-k S^{-1}\right) S_{k}^{-1} A_{k}^{-1}-\left( S-k S^{-1}+R^{\prime } \psi ^{-1} R\right) \right] A_{k} \\&\quad =A_{k}\left[ \left( S_{k}+R^{\prime } \psi ^{-1} R\right) S_{k}^{-1}NS_{k}^{-1}\left( S_{k}+R^{\prime } \psi ^{-1} R\right) -\left( N+R^{\prime } \psi ^{-1} R\right) \right] A_{k} \\&\quad =A_{k}\left[ \left( S_{k}+Q\right) S_{k}^{-1} N S_{k}^{-1}\left( S_{k}+Q\right) -(N+Q)\right] A_{k} \\&\quad =A_{k}\left[ \left( I+Q S_{k}^{-1}\right) N\left( I+S_{k}^{-1} Q\right) -(N+Q)\right] A_{k} \\&\quad =A_{k}\left[ N+N S_{k}^{-1} Q+Q S_{k}^{-1} N+Q S_{k}^{-1} N S_{k}^{-1} Q-(N+Q)\right] A_{k} \\&\quad =A_{k}\left( N S_{k}^{-1} Q+Q S_{k}^{-1} N+Q S_{k}^{-1} N S_{k}^{-1} Q-Q\right) A_{k} \\&\quad =A_{k} B A_{k}, \end{aligned}$$

where$$N=S-kS^{-1}, Q=R^{\prime } \psi ^{-1} R, B=N S_{k}^{-1} Q+Q S_{k}^{-1} N+Q S_{k}^{-1} N S_{k}^{-1} Q-Q$$

According to the Lemma 3.1, it can be obtained that if $k<\min \nolimits _{i=1}^{p}\lambda _{i}^{2}$, then $N>0$. So $B>0$ if and only if $k<\min \nolimits _{i=1}^{p}\lambda _{i}^{2},\lambda _{1} Q\left( N S_{k}^{-1} Q+Q S_{k}^{-1} N+Q S_{k}^{-1} N S_{k}^{-1} Q\right) ^{-1}<1$.

As long as $k<\min \nolimits _{i=1}^{p}\lambda _{i}^{2},\lambda _{1} Q\left( N S_{k}^{-1} Q+Q S_{k}^{-1} N+Q S_{k}^{-1} N S_{k}^{-1} Q\right) ^{-1}<1$, following conclusions can be obtained:

$$\begin{aligned} \Delta _{2} \ge 0 \text{ if } \text{ and } \text{ only } \text{ if } b_{1}^{\prime }\left( \sigma ^{2} A_{k} B A_{k}+b_{2} b_{2}^{\prime }\right) ^{-1} b_{1} \le 1 . \text{ Therefore, } \text{ there } \text{ is } \text{ Theorem } 3.2. \end{aligned}$$

#### Theorem 3.3

*When* $k<\min \limits _{i=1}^{p}\lambda _{i}^{2},\lambda _{1} Q\left( N S_{k}^{-1} Q+Q S_{k}^{-1} N+Q S_{k}^{-1} N S_{k}^{-1} Q\right) ^{-1}<1$, *the necessary and sufficient conditions for mixed KL estimator* $\hat{\beta }_{MKL}$ *to be superior to KL estimator* $\hat{\beta }_{KL}$ *under MSEM criterion are as follows:*

25 $$\begin{aligned} b_{1}^{\prime }\left( \sigma ^{2} A_{k} B A_{k}+b_{2} b_{2}^{\prime }\right) ^{-1} b_{1} \le 1 \end{aligned}$$

### Comparison between mixed KL estimator and OLS estimator

From Eqs. (16) and (19), we make

26 $$\begin{aligned} \Delta _{3}= & {} {\text {MSEM}}\left( \hat{\beta }_{OLS}\right) -{\text {MSEM}}\left( \hat{\beta }_{MKL}\right) \nonumber \\= & {} \sigma ^{2} S^{-1}-\sigma ^{2} A_{k}\left( X^{\prime } X-k S^{-1}+R^{\prime } \psi ^{-1} R\right) A_{k}-b_{1} b_{1}^{\prime }\nonumber \\= & {} \sigma ^{2}\left[ S^{-1}-A_{k}\left( X^{\prime } X-k S^{-1}+R^{\prime } \psi ^{-1} R\right) A_{k}\right] -b_{1} b_{1}^{\prime } \end{aligned}$$

Because

$$\begin{aligned}&S^{-1}-A_{k}\left( X^{\prime } X-k S^{-1}+R^{\prime } \psi ^{-1} R\right) A_{k}\\&\quad =A_{k} A_{k}^{-1} S^{-1} A_{k}^{-1} A_{k}-A_{k}\left( X^{\prime } X-k S^{-1}+R^{\prime } \psi ^{-1} R\right) A_{k} \\&\quad =A_{k}\left[ A_{k}^{-1} S^{-1} A_{k}^{-1}-\left( X^{\prime } X-k S^{-1}+R^{\prime } \psi ^{-1} R\right) \right] A_{k} \\&\quad =A_{k}\left[ \left( S+k I+Q\right) S^{-1}\left( S+k I+Q\right) -\left( S-k S^{-1}+Q\right) \right] A_{k} \\&\quad =A_{k}\left[ \left( I+k S^{-1}+QS^{-1}\right) \left( S+k I+Q\right) -\left( S-k S^{-1}+Q\right) \right] A_{k} \\&\quad =A_{k}\left[ S+k I+Q+\left( I+k S^{-1}+QS^{-1}\right) \left( k I+Q\right) -\left( S-k S^{-1}+Q\right) \right] A_{k} \\&\quad =A_{k}\left[ k I+k S^{-1}+\left( I+k S^{-1}+QS^{-1}\right) \left( k I+Q\right) \right] A_{k} \\&\quad =A_{k}\left( 2 k I+k S^{-1}+k^{2} S^{-1}+Q+k S^{-1} Q+k Q S^{-1}+Q S^{-1} Q\right) A_{k} \\&\quad =A_{k}\left[ 2 k I+k S^{-1}+k^{2} S^{-1}+Q+k\left( S^{-1} Q+Q S^{-1}\right) +Q S^{-1} Q\right] A_{k} \\&\quad =A_{k}\left[ 2 k I+k S^{-1}+k^{2} S^{-1}+Q+k C+Q S^{-1} Q\right] A_{k} \end{aligned}$$

where $$C=S^{-1} Q+Q S^{-1}$$.

Because $C=C^{\prime }$, and $\lambda _{i}\left( S^{-1} Q\right) =\lambda _{i}\left( S^{-\frac{1}{2}} Q S^{-\frac{1}{2}}\right) >0$, we can get $C>0$, so $2 k I+k S^{-1}+k^{2} S^{-1}+Q+k C+Q S^{-1} Q>0$, that is $S^{-1}-A_{k}\left( X^{\prime } X-k S^{-1}+R^{\prime } \psi ^{-1} R\right) A_{k}>0$, Theorem 3.4 is obtained.

#### Theorem 3.4

*The necessary and sufficient conditions for mixed KL estimator* $\hat{\beta }_{MKL}$ *to be superior to* $\hat{\beta }_{OLS}$ *under MSEM criterion are as follows:*

27 $$\begin{aligned} \sigma ^{-2} b_{1}^{\prime }\left[ S^{-1}-A_{k}\left( X^{\prime } X-k S^{-1}+R^{\prime } \psi ^{-1} R\right) A_{k}\right] b_{1} \le 1 \end{aligned}$$

### Comparison between mixed KL estimator and mixed ridge estimator

From Eqs. (16) and (22), we make

28 $$\begin{aligned} \Delta _{4}= & {} {\text {MSEM}}\left( \beta _{M R E}\right) -{\text {MSEM}}\left( \beta _{M K L}\right) \nonumber \\= & {} \sigma ^{2} A_{k}\left( S+Q\right) A_{k}+k^{2} A_{k} \beta \beta ^{\prime } A_{k}-\sigma ^{2} A_{k}\left( S-k S^{-1}+Q\right) A_{k}-4 k^{2} A_{k} \beta \beta ^{\prime } A_{k}\nonumber \\= & {} \sigma ^{2} A_{k} M A_{k}-\sigma ^{2} A_{k}\left( M-k S^{-1}\right) A_{k}-3 k^{2} A_{k} \beta \beta ^{\prime } A_{k}\nonumber \\= & {} \sigma ^{2} A_{k}\left[ M-\left( M-k S^{-1}\right) \right] A_{k}-3 k^{2} A_{k} \beta \beta ^{\prime } A_{k}\nonumber \\= & {} k \sigma ^{2} A_{k} S^{-1} A_{k}-3 k^{2} A_{k} \beta \beta ^{\prime } A_{k}\nonumber \\= & {} k A_{k}\left( \sigma ^{2} S^{-1}-3 k \beta \beta ^{\prime }\right) A_{k} \end{aligned}$$

#### Theorem 3.5

*The necessary and sufficient conditions for mixed KL estimator* $\hat{\beta }_{MKL}$ *to be superior to the mixed ridge estimator* $\hat{\beta }_{MRE}$ *under MSEM criterion are as follows:*

29 $$\begin{aligned} 3 k \sigma ^{-2} \beta ^{\prime } S \beta \le 1 \end{aligned}$$.

## Numerical example and simulation study

In order to further explain the theoretical results, this section will verify and analyze the above theoretical results through examples.

The example analysis data adopts the percentage data of research and development expenses in GNP of several countries from 1972 to 1986 used by Gruber^21^, Akdeniz and Erol^22^, in which $x_{1}$ represents France, $x_{2}$ represents West Germany, $x_{3}$ represents Japan, $x_{4}$ represents the former Soviet Union and *y* represents the United States. See Table 1 for specific data.

**Table 1:** 1972–1986 research and development expenditure as a percentage of GNP.

Year	$x_{1}$	$x_{2}$	$x_{3}$	$x_{4}$	*y*
1972	1.9	2.2	1.9	3.7	2.3
1975	1.8	2.2	2	3.8	2.2
1979	1.8	2.4	2.1	3.6	2.2
1980	1.8	2.4	2.2	3.8	2.3
1981	2	2.5	2.3	3.8	2.4
1982	2.1	2.6	2.4	3.7	2.5
1983	2.1	2.6	2.6	3.8	2.6
1984	2.2	2.6	2.6	4	2.6
1985	2.3	2.8	2.8	3.7	2.7
1986	2.3	2.7	2.8	3.8	2.7

The data in Table 1 are processed as follows

$$\begin{aligned} X=\left( \begin{array}{cccc} 7 &{} 26 &{} 6 &{} 60 \\ 1 &{} 29 &{} 15 &{} 52 \\ 11 &{} 56 &{} 8 &{} 20 \\ 11 &{} 31 &{} 8 &{} 47 \\ 7 &{} 52 &{} 6 &{} 33 \\ 11 &{} 55 &{} 9 &{} 22 \\ 3 &{} 71 &{} 17 &{} 6 \\ 1 &{} 31 &{} 22 &{} 44 \\ 2 &{} 54 &{} 18 &{} 22 \\ 21 &{} 47 &{} 4 &{} 26 \\ 1 &{} 40 &{} 23 &{} 34 \\ 11 &{} 66 &{} 9 &{} 12 \\ 10 &{} 68 &{} 12 &{} 12 \end{array}\right) , y=\left( \begin{array}{c} 78.5 \\ 74.3 \\ 104.3 \\ 87.6 \\ 95.9 \\ 109.2 \\ 102.7 \\ 72.5 \\ 93.1 \\ 115.9 \\ 83.8 \\ 113.3 \\ 109.4 \end{array}\right) \end{aligned}$$

Firstly, it is easy to calculate that the eigenvalue of $X^{\prime } X$ is $\lambda _{1}=302.9626$, $\lambda _{2}=0.7283$, $\lambda _{3}=0.0446, \lambda _{4}=0.0345$,the OLS estimator of $\sigma ^{2}$ is $\hat{\sigma }^{2}=0.0015$, and $\mathrm {OLS}$ estimator of $\beta$ is $\hat{\beta }_{O L S}=(0.6455,0.0896,0.1436,0.1526)^{\prime }$.

We can use the method proposey by Kibria and Lukman^14^ to choose the biasing parameter *k*, and we can also use the generalized cross validation (GCV) criterion and the cross validation (CV) to choose the biasing parameter, the reference can refer to Arashi et al.^23^, Roozbeh^24^, and Roozbeh et al.^25^. In this paper we use the method propose by Kibria and Lukman^14^ to choose the biasing parameter *k*, which is given as follows:

30 $$\begin{aligned} \hat{k}_{i}=\frac{\hat{\sigma }^{2}}{2\hat{\alpha _{i}}^{2}+\left( \hat{\sigma }^{2} / \lambda _{i}\right) } \end{aligned}$$

we take $$k=\hat{k}_{\min }$$.

Consider the following stochastic restrictions, this can refer to Roozbeh et al.^26^ and Roozbeh and Hamzah^27^:

$$\begin{aligned} r=R \beta +e, R=\left( \begin{array}{llll} 1&-2&-2&-2 \end{array}\right) , r=1, e \sim \left( 0, \hat{\sigma }^{2}\right) \end{aligned}$$

For the mixed estimator, KL estimator, OLS estimator, mixed ridge estimator and mixed KL estimator proposed in this paper. The MSE is presented in Table 2.

**Table 2:** Estimated MSE.

	$\hat{\beta }_{ME}$	$\hat{\beta }_{KL}$	$\hat{\beta }_{O L S}$	$\hat{\beta }_{MRE}$	$\hat{\beta }_{M K L}$
$\alpha _{1}$	0.6455	0.6102	0.6455	0.6452	0.6449
$\alpha _{2}$	0.0896	0.0988	0.0896	0.0894	0.0892
$\alpha _{3}$	0.1436	0.1577	0.1436	0.1434	0.1433
$\alpha _{4}$	0.1526	0.1566	0.1526	0.1530	0.1534
${\text {MSE}}$	0.0431	0.0235	0.0561	0.0390	0.0180

As can be seen from Table 2:

When *k* takes $\hat{k}_{\min }=0.018$, the MSE value of mixed KL estimator $\hat{\beta }_{M K L}$ is better than that of mixed estimator, KL estimator,OLS estimator and mixed ridge estimator. Consistent with the theoretical results of this paper, it can be concluded that adding stochastic restrictions may have better estimation effect under certain conditions. So in practice we can use the stochastic restrictions to address the multicollinearity.

Next, we consider Monte Carlo simulation analysis.

Firstly, the generation of relevant parameters and data in the process of simulation analysis is briefly described.

The data generation of explanatory variables adopts the same method as McDonald and Galarneau^28^, Gibbons^29^), that is, it is generated by the following equation:

$$\begin{aligned} x_{i j}=\left( 1-\rho ^{2}\right) ^{1 / 2} z_{i j}+\rho z_{i p}, \quad i=1,2, \ldots , n, \quad j=1,2, \ldots , p \end{aligned}$$

where $z_{i j}$ is the random number generated by the standard normal random variable, $\rho$ is the given constant, and $\rho ^{2}$ theoretically represents the correlation between two different variables, so $\rho ^{2}$ reflects the degree of complex collinearity of the model to some extent. In this simulation analysis, we consider three cases $\rho =0.85,0.9,0.99$, set $$p=3, r=1, R=\left( \begin{array}{lll}1&-2&-2\end{array}\right) , e \sim (0, \sigma ^{2}),n=30,50,70,100$$.

For a given design matrix *X*, we take the orthogonalized eigenvector corresponding to the maximum eigenvalue of $X^{\prime } X$ as the real value of parameter vector $\beta$.

The data corresponding to the response variable is generated by the following equation:

$$\begin{aligned} y_{i}=\beta _{1} x_{i 1}+\beta _{2} x_{i 2}+\ldots +\beta _{p} x_{i p}+\varepsilon _{i}, i=1,2, \ldots , n \end{aligned}$$

where $\varepsilon _{i}$ is the mean of zero, and random vector with variance of $\sigma ^{2}=0.1,1,5,10$.

See Tables 3, 4, 5, 6, 7, 8, 9, 10, 11, 12, 13, 14, 15, 16, 17 and 18 for simulation analysis and calculation results.

**Table 3:** Estimated MSE when $\sigma ^{2}=0.1, n=30$.

$\rho$	$\hat{\beta }_{ME}$	$\hat{\beta }_{KL}$	$\hat{\beta }_{O L S}$	$\hat{\beta }_{MRE}$	$\hat{\beta }_{M K L}$
0.85	0.0024	0.0028	0.0028	0.0023	0.0023
0.9	0.0032	0.0042	0.0043	0.0032	0.0032
0.99	0.0197	0.0214	0.0282	0.0185	0.0160

**Table 4:** Estimated MSE when $\sigma ^{2}=0.1, n=50$.

$\rho$	$\hat{\beta }_{ME}$	$\hat{\beta }_{KL}$	$\hat{\beta }_{O L S}$	$\hat{\beta }_{MRE}$	$\hat{\beta }_{M K L}$
0.85	0.0012	0.0013	0.0013	0.0012	0.0012
0.9	0.0027	0.0031	0.0031	0.0027	0.0027
0.99	0.0146	0.0221	0.0245	0.0158	0.0152

**Table 5:** Estimated MSE when $\sigma ^{2}=0.1, n=70$.

$\rho$	$\hat{\beta }_{ME}$	$\hat{\beta }_{KL}$	$\hat{\beta }_{O L S}$	$\hat{\beta }_{MRE}$	$\hat{\beta }_{M K L}$
0.85	0.0011	0.0011	0.0011	0.0011	0.0011
0.9	0.0016	0.0019	0.0020	0.0016	0.0016
0.99	0.0126	0.0179	0.0205	0.0119	0.0015

**Table 6:** Estimated MSE when $\sigma ^{2}=0.1, n=100$.

$\rho$	$\hat{\beta }_{ME}$	$\hat{\beta }_{KL}$	$\hat{\beta }_{O L S}$	$\hat{\beta }_{MRE}$	$\hat{\beta }_{M K L}$
0.85	0.0009	0.0009	0.0009	0.0009	0.0009
0.9	0.0015	0.0018	0.0018	0.0015	0.0015
0.99	0.0104	0.0137	0.0142	0.0102	0.0101

**Table 7:** Estimated MSE when $\sigma ^{2}=1, n=30$.

$\rho$	$\hat{\beta }_{ME}$	$\hat{\beta }_{KL}$	$\hat{\beta }_{O L S}$	$\hat{\beta }_{MRE}$	$\hat{\beta }_{M K L}$
0.85	0.2375	0.1963	0.2710	0.1752	0.1697
0.9	0.3463	0.2594	0.4260	0.2354	0.2231
0.99	3.5782	2.7915	4.2485	1.8076	1.3056

**Table 8:** Estimated MSE when $\sigma ^{2}=1, n=50$.

$\rho$	$\hat{\beta }_{ME}$	$\hat{\beta }_{KL}$	$\hat{\beta }_{O L S}$	$\hat{\beta }_{MRE}$	$\hat{\beta }_{M K L}$
0.85	0.1908	0.1775	0.2012	0.1673	0.1675
0.9	0.1932	0.1968	0.2340	0.1662	0.1660
0.99	1.9234	1.3309	2.9377	1.3809	0.7301

**Table 9:** Estimated MSE when $\sigma ^{2}=1, n=70$.

$\rho$	$\hat{\beta }_{ME}$	$\hat{\beta }_{KL}$	$\hat{\beta }_{O L S}$	$\hat{\beta }_{MRE}$	$\hat{\beta }_{M K L}$
0.85	0.0984	0.0868	0.1033	0.0835	0.0828
0.9	0.1524	0.1533	0.1906	0.1277	0.1270
0.99	1.7543	1.1738	2.0825	1.0379	0.9163

**Table 10:** Estimated MSE when $\sigma ^{2}=1, n=100$.

$\rho$	$\hat{\beta }_{ME}$	$\hat{\beta }_{KL}$	$\hat{\beta }_{O L S}$	$\hat{\beta }_{MRE}$	$\hat{\beta }_{M K L}$
0.85	0.0751	0.0737	0.0823	0.0680	0.0678
0.9	0.1424	0.1314	0.1548	0.1218	0.1215
0.99	1.0463	0.4883	1.3303	0.5769	0.3634

**Table 11:** Estimated MSE when $\sigma ^{2}=5, n=30$.

$\rho$	$\hat{\beta }_{ME}$	$\hat{\beta }_{KL}$	$\hat{\beta }_{O L S}$	$\hat{\beta }_{MRE}$	$\hat{\beta }_{M K L}$
0.85	8.7289	3.3534	13.8404	3.7486	3.3308
0.9	10.7412	4.5196	13.4709	4.4088	3.7785
0.99	84.6872	115.8703	143.0686	45.0361	31.8312

**Table 12:** Estimated MSE when $\sigma ^{2}=5, n=50$.

$\rho$	$\hat{\beta }_{ME}$	$\hat{\beta }_{KL}$	$\hat{\beta }_{O L S}$	$\hat{\beta }_{MRE}$	$\hat{\beta }_{M K L}$
0.85	6.3491	2.8390	7.8722	2.7472	2.5652
0.9	4.3795	2.1116	4.8651	1.8364	1.7838
0.99	51.4274	44.6585	75.3850	30.2169	24.9511

**Table 13:** Estimated MSE when $\sigma ^{2}=5, n=70$.

$\rho$	$\hat{\beta }_{ME}$	$\hat{\beta }_{KL}$	$\hat{\beta }_{O L S}$	$\hat{\beta }_{MRE}$	$\hat{\beta }_{M K L}$
0.85	3.2597	1.7710	3.4194	1.3309	1.5452
0.9	3.9034	1.7501	4.3951	1.6719	1.5983
0.99	27.7983	32.8762	38.5172	21.0908	19.3660

**Table 14:** Estimated MSE when $\sigma ^{2}=5, n=100$.

$\rho$	$\hat{\beta }_{ME}$	$\hat{\beta }_{KL}$	$\hat{\beta }_{O L S}$	$\hat{\beta }_{MRE}$	$\hat{\beta }_{M K L}$
0.85	0.8875	1.7599	0.9286	1.9500	0.8946
0.9	1.4590	2.9916	1.5563	3.5008	1.4880
0.99	10.8178	22.8642	19.2461	31.9632	11.7280

**Table 15:** Estimated MSE when $\sigma ^{2}=10, n=30$.

$\rho$	$\hat{\beta }_{ME}$	$\hat{\beta }_{KL}$	$\hat{\beta }_{O L S}$	$\hat{\beta }_{MRE}$	$\hat{\beta }_{M K L}$
0.85	23.4609	8.7136	27.9864	8.4935	8.0303
0.9	28.7442	13.2491	31.8223	12.4392	11.8325
0.99	343.6973	450.0098	539.6959	250.2456	106.987

**Table 16:** Estimated MSE when $\sigma ^{2}=10, n=50$.

$\rho$	$\hat{\beta }_{ME}$	$\hat{\beta }_{KL}$	$\hat{\beta }_{O L S}$	$\hat{\beta }_{MRE}$	$\hat{\beta }_{M K L}$
0.85	19.1189	7.0762	23.1833	6.7891	6.8042
0.9	30.4727	10.6969	32.8809	10.1258	9.0393
0.99	226.4676	335.4425	390.911	130.0564	97.6481

**Table 17:** Estimated MSE when $\sigma ^{2}=10, n=70$.

$\rho$	$\hat{\beta }_{ME}$	$\hat{\beta }_{KL}$	$\hat{\beta }_{O L S}$	$\hat{\beta }_{MRE}$	$\hat{\beta }_{M K L}$
0.85	11.7984	3.8394	13.2484	3.9206	3.7376
0.9	18.0514	5.6690	19.9073	5.7731	5.4011
0.99	197.0861	114.1176	237.743	91.9548	54.3546

**Table 18:** Estimated MSE when $\sigma ^{2}=10, n=100$.

$\rho$	$\hat{\beta }_{ME}$	$\hat{\beta }_{KL}$	$\hat{\beta }_{O L S}$	$\hat{\beta }_{MRE}$	$\hat{\beta }_{M K L}$
0.85	8.6620	3.0434	8.9405	3.1080	2.9602
0.9	16.3215	5.6973	17.6756	5.6112	5.4424
0.99	120.1565	67.5786	168.6498	62.5914	47.1027

Based on Tables 3, 4, 5, 6, 7, 8, 9, 10, 11, 12, 13, 14, 15, 16, 17 and 18, the following conclusions are drawn:

1. The mean square error of all estimates increases with the increase of $\rho$ and decreases with the increase of *n*; 
2. The new estimator mixed KL estimator always has the minimum MSE when all given *n* and $\sigma ^{2}$ ,and *k* takes $\hat{k}_{\min }$. Consistent with the results of Theorems 3.2–3.5 in this paper, under certain conditions, mixed KL estimator $\hat{\beta }_{MKL}$ is better than mixed estimator $\hat{\beta }_{M E}$, KL estimator $\hat{\beta }_{K L}$, least square estimator $\hat{\beta }_{OLS}$ and mixed ridge estimator $\hat{\beta }_{M R E}$ under MSE criterion;
3. Under the same conditions, mixed estimator $\hat{\beta }_{ME}$,mixed ridge estimator $\hat{\beta }_{MRE}$ and mixed KL estimator $\hat{\beta }_{MKL}$ are better than unconstrained least squares estimator $\hat{\beta }_{OLS}$ under MSE criterion, mixed KL estimator $\hat{\beta }_{M K L}$ is better than unconstrained KL estimator $\hat{\beta }_{K L}$ under MSE criterion.

## Conclusions

In this paper, a new mixed KL estimator considering the prior information about parameters in sample information in linear model is proposed, and the properties of the new estimator are discussed. The necessary and sufficient conditions for KL estimator to be better than mixed estimator, KL estimator, OLS estimator and mixed ridge estimator under the criterion of mean square error matrix are given, and the proofs are given respectively. Then the theoretical results are verified by examples and simulation analysis.
